# Clinical study on hemorrhagic complications in antiplatelet and anticoagulated patients undergoing dental treatment

**DOI:** 10.4317/medoral.27715

**Published:** 2025-11-22

**Authors:** Carmen López-Carriches, Ricardo Taheri, Cristina Madrigal-Martínez-Pereda

**Affiliations:** 1 Department of Dental Clinic Specialties. School of Dentistry. Universidad Complutense de Madrid. Spain; 2 School of Dentistry, Universidad Complutense of Madrid. Spain

## Abstract

**Background:**

Patients undergoing treatment with antiplatelet or anticoagulant drugs are attending dental consultations. Therefore, it has become essential to evaluate the patient before treatment and to conduct a thorough medical history. The goal of this study is to analyze the patients attending a dental clinic over a period of 6 months to determine how many are taking antiplatelet or anticoagulant medications, what dental treatment they seek, what hemostatic measures are taken, and whether they suffer hemorrhagic complications as a result of the treatment. This will help to prevent such complications by applying appropriate protocols, especially in the field of oral surgery.

**Material and Methods:**

Over a six-month period, 867 patients attended a dental clinic in Madrid. Of these, 43 were taking antiplatelet agents or anticoagulants. A descriptive statistical analysis was conducted to gather data on age, gender, medication, dental treatment received, and hemorrhagic complications.

**Results:**

Only 4.9% of the patients attending the dental clinic were taking antiplatelet or anticoagulant medications. Specifically, 53.48% were taking antiplatelet agents, 34.8% were taking direct oral anticoagulants, and 11.62% were on vitamin K antagonist anticoagulants. After applying the appropriate clinical protocol in each case, only one patient experienced postoperative bleeding, which was not severe.

**Conclusions:**

Based on the results, it may not be necessary to withdraw antiplatelet or anticoagulant therapy due to the low incidence and non-severity of complications in simple dental procedures.

## Introduction

With the aging of the population, more and more patients undergoing treatment with antiplatelet or anticoagulant drugs are attending dental consultations.

Therefore, it has become essential to evaluate the patient before treatment and to conduct a thorough medical history. The HAS-BLED scale is recommended to be applied to assess the bleeding risk in patients who are candidates for oral anticoagulants. (H: Hypertension, A: Abnormal kidney and/or liver function, S: Stroke, B: Bleeding, L: Labile INR, E: Elderly, D: Drugs and/or alcohol). Patients with a score higher than 3 points may have a greater risk of bleeding. (1 , 2) The treatment protocols for these patients are under continuous review. Not long ago, clinical protocols recommended to withdraw Adiro (acetylsalicylic acid) for seven days. However, that recommendation began to be reconsidered in order to avoid the risk of thromboembolism. (3) It is important to remember many patients with cardiovascular disease are on anticoagulants, either direct oral anticoagulants or vitamin K antagonists, or antiplatelet agents. Clinicians must be aware of the risk of hemorrhage and the appropriate treatment protocols for these patients. The objective of this study is to determine whether patients taking antiplatelet or anticoagulant medications and undergoing dental treatment experience hemorrhagic complications. Secondly, to establish guidelines for treating these patients in order to minimize the risk of thromboembolism.

## Material and Methods

A descriptive observational study was conducted involving 867 patients who attended a dental clinic over a six-month period. There were no exclusion criteria. A spreadsheet was created to record each patient's history number, age, gender, medications used, and the dental treatment received.

A total of 43 patients were identified for taking antiplatelet or anticoagulant medications. The management of these medications prior to dental treatment was also noted:

Adiro was continued without interruption. Sintrom (acenocoumarol): An INR test was performed on the day of the procedure, and no adjustments were made if the value was below 3. Direct oral anticoagulants: Surgery was scheduled in the morning to allow for at least 24 hours without medication. For patients on a once-daily regimen, the dose was not withheld. For those on a twice-daily regimen, the evening dose prior to surgery was omitted, and treatment was resumed a few hours after the procedure.

Anti-hemorrhagic measures used during the procedure, and any postoperative hemorrhage or hematoma were also documented.

A descriptive statistical analysis was conducted based on the data collected. The analysis included the calculation of measures of central tendency (mean, median) and dispersion for continuous variables, as well as frequency distribution and percentages for categorical variables. The analysis was conducted using the Python programming language.

A literature review of the past 10 years was conducted using the PubMed Medline and The Cochrane Library Plus databases, targeting articles related to patients undergoing oral surgery while on anticoagulant or antiplatelet therapy.

This report presents an exploratory data analysis of the patients' dataset. This dataset includes patient demographics, antiaggregant and anticoagulant use, treatment types, hemorrhage occurrences, and hemostasis measures.

## Results

Demographic characteristics of the study population

**Figure 1:**
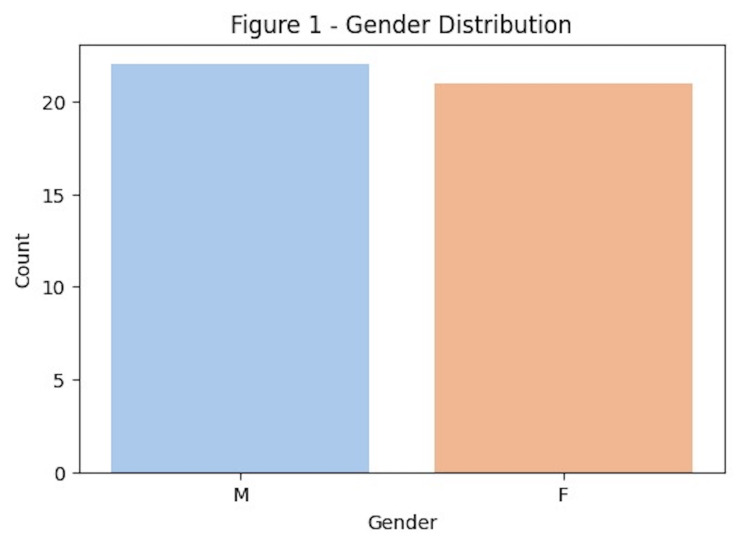
Gender distribution of patients.

A total of 43 patients were included in the study. Among all patients attending the clinic, 4.9% were receiving antiplatelet or anticoagulant therapy. 21 were females and 22 were males. Therefore, 49% of the patients were females and 51% males. (Figure 1)

1

**Figure 2:**
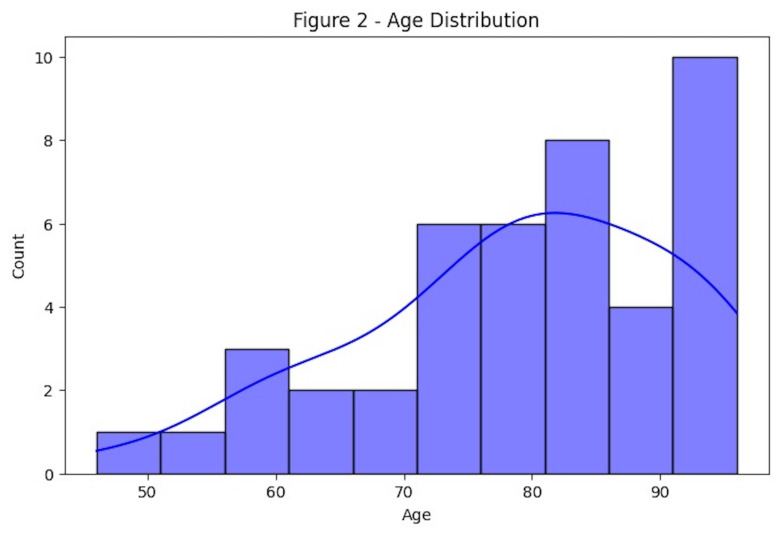
Age distribution of patients.

Patient age ranged from 46 to 96 years, with a mean of 79.07. The distribution was right-skewed, with the majority of patients aged 70 or older (Figure 2), highlighting the need for specialized care tailored to an aging population.

2

**Figure 3:**
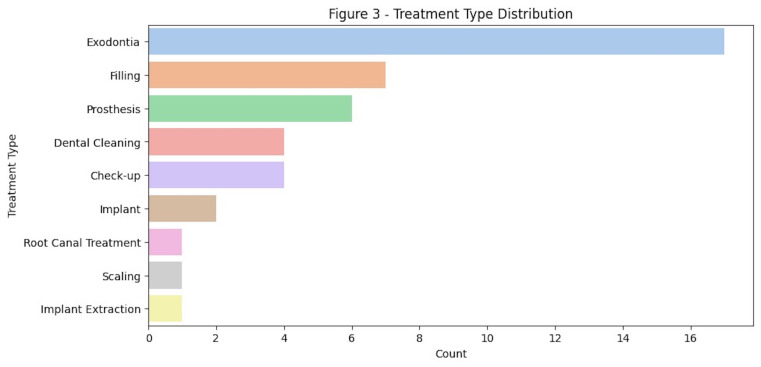
Most common treatments.

The dataset revealed that the most frequent treatments performed in the clinic include extractions, composite restorations and prosthetic procedures. (Figure 3).

3

**Figure 4:**
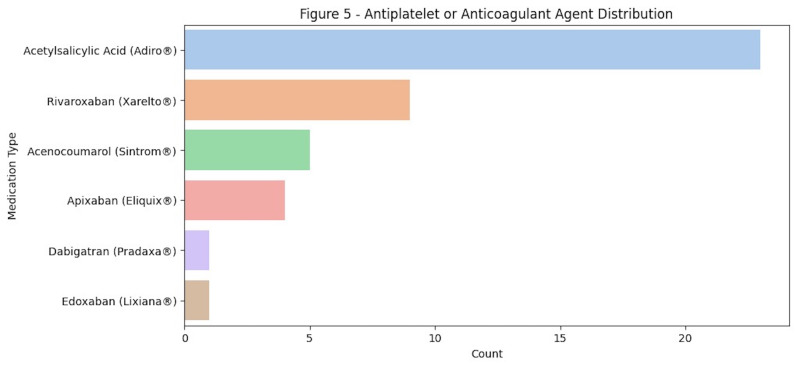
Most common anticoagulant antiplatelet used.

The most commonly used medication among patients was Adiro, followed by Xarelto and Sintrom, as shown in Figure 4.

4

Hemorrhage occurred in only one case, involving a complex extraction of a mandibular root remnant. The patient was on Sintrom, with an INR of 2.5 measured on the day of the procedure. Hemostasis was achieved through re-suturing and the application of Amchafibrin. Additionally, two patients developed hematomas following dental procedures. Hematomas, although generally less severe than active hemorrhage, may indicate underlying coagulation disorders or inadequate hemostatic control. One of these patients underwent a complex tooth extraction while being treated with Sintrom, and the other received a dental implant while on Eliquis.

## Discussion

A comprehensive medical history will allow us to understand why the patient is on antiplatelet or anticoagulant therapy, as well as their risk of hemorrhage or thromboembolism.

Most treatments in the dataset did not result in hemorrhage. These procedures varied in complexity, with some requiring post-treatment hemostatic interventions.

Adiro has a half-life of 5-7 days, and its withdrawal one or two days before surgery is not recommended. However, the treating physician may choose to prescribe the lowest effective dose-typically 100 mg instead of 300 mg-several days prior to the procedure. For minor interventions, such as simple extractions (fewer than three teeth) or uncomplicated surgeries (e.g., implants without grafting), discontinuation of antiplatelet therapy is not necessary. Local hemostatic measures should be implemented. The risk of discontinuing antiplatelet therapy generally outweighs the risk of bleeding. (4)

In patients with dual antiplatelet therapy, there are studies that suggest that excessive bleeding may be prevented by local measures of haemostasis in simple tooth extractions. (5) In the study conducted by Lillis et al (6) those who bled the most were under dual therapy, but there were no differences with the controls in the case of Adiro alone or Clopidogrel alone. Therefore, there are several authors who consider that it is not necessary to interrupt antiplatelet treatment in dental extractions. Although, they found greater bleeding with dual treatment and with Clopidogrel versus Adiro. However, in case of bleeding, it is satisfactorily resolved with local hemostatic measures. (7)

Our recommendation is to avoid performing extractions in patients receiving dual antiplatelet therapy; instead, we prefer to wait until the cardiologist discontinues one of the medications. However, no such patients were included in our study

Also, patients treated with Sintrom should not receive modifications or suspension of their treatment. However, the patient must be asked for an INR of the same day or the day before the procedure. If the value is under 3, surgery can be performed. (8 , 9)

Moreover, if the value of INR is above 3 and an important bleeding is expected due to procedures such as multiple extractions, multiple implants or a flap surgery combined with ostectomy. In these cases, the dose must be adjusted or the Sintrom should be changed to heparin by the treating physician two days prior surgery and resumed the same day as the extraction- about 12 hours after. Also, the patient must continue with heparin treatment until the anticoagulation levels are reached.

Patients may also be taking direct oral anticoagulants (DOACs), which include the following:

Dabigatran, a thrombin inhibitor, with a recommended dosage of 150 mg twice daily. (10 , 11) Rivaroxaban, a direct Factor Xa inhibitor, recommended at 15-20 mg once daily. (12) Apixaban, another Factor Xa inhibitor, typically prescribed at 5 mg twice daily. (13) Edoxaban, also a Factor Xa inhibitor, with a recommended dosage of 60 mg once daily. (14)

The time to reach maximum plasma concentration ranges from 1 to 4 hours, and the anticoagulant effect usually diminishes within 12 to 24 hours after administration. (13)

For non-invasive oral surgeries, current recommendations advise against interrupting anticoagulant therapy to reduce the risk of thromboembolism. (14) In our clinical protocol, the evening dose-if applicable-is suspended, always in consultation with the patient's physician, as surgical procedures can become complicated.

For more invasive surgical procedures-such as multiple extractions, implant placement with grafting, wide mucoperiosteal flaps, or impacted third molar removal involving ostectomy-it is essential to evaluate both bleeding and thrombotic risks. Treatment should be scheduled early in the day. For patients taking Apixaban or Dabigatran (administered twice daily), some clinicians recommend that the morning dose should be omitted. This contrasts with our protocol, which suspends the evening dose, achieving a similar 24-hour drug-free window. Patients taking Rivaroxaban or Edoxaban (once daily) should delay the dose until at least four hours after the procedure, which aligns with our current approach. If the usual dosing time is in the afternoon, no adjustment is necessary. (9 , 15)

According to Manfredi et al, (16) there are no differences found in postoperative bleeding in patients who suspend treatment with direct oral anticoagulants compared to those who did not suspend it.

Many studies support the recommendation of not removing direct anticoagulants before simple extractions. (17 , 18 , 19) The procedure must be performed as late as possible after the last intake (at least 12 hours after in twice daily dose and 24 hours after in single daily dose). (20)

The use of hemostatic measures during or after the procedure can often eliminate the need to suspend antiplatelet or anticoagulant therapy. The most recommended techniques include alveolar curettage to remove granulation tissue, use of sutures (preferably resorbable), gauze compression, fibrin sponges, resorbable gelatin sponges, oxidized cellulose, and bone wax.

Antifibrinolytic agents may also be employed, such as Tranexamic acid (500 mg injectable solution), which can be used to soak gauze pads for gentle rinsing during the first two days-at least one minute every 6 hours. Also, Epsilon-aminocaproic acid (4 g injectable solution), may be applied in the same manner as Tranexamic acid. (21 , 22)

In our study, hemostatic techniques such as suturing and the use of fibrin sponges were routinely applied to control and minimize excessive bleeding.

As a last resort, in cases where bleeding persists despite standard measures in patients taking vitamin K antagonists, oral vitamin K (1-2 mg) may be administered to reverse the anticoagulant effect and reduce INR (21). There are also reversal agents available for direct oral anticoagulants, which are indicated in cases of life-threatening hemorrhage. (23 , 24)

The patient is given written postoperative instructions and it is advisable to keep the patient in the waiting room for a period following the procedure to confirm the absence of bleeding, and to follow up with a phone call later that day and the next. In this way we were able to solve the only case of hemorrhage, avoiding the patient having to go to the hospital emergency room. Although such hemorrhages are generally not severe, they often cause significant concern for patients, especially when occurring in the oral cavity.

## Conclusions

The current recommendations that we have found in the reviewed articles is not to withdraw the antiplatelet/anticoagulant therapy. However, the studies referred to simple extraction procedures or those with a moderate risk of bleeding. The recommended protocol should include local measures of hemostasis.

## Data Availability

Declared none.
